# Epitope Tags beside the N-Terminal Cytoplasmic Tail of Human BST-2 Alter Its Intracellular Trafficking and HIV-1 Restriction

**DOI:** 10.1371/journal.pone.0111422

**Published:** 2014-10-27

**Authors:** Mingyu Lv, Jiawen Wang, Jingyao Zhang, Biao Zhang, Xiaodan Wang, Yingzi Zhu, Tao Zuo, Donglai Liu, Xiaojun Li, Jiaxin Wu, Haihong Zhang, Bin Yu, Hui Wu, Xinghong Zhao, Wei Kong, Xianghui Yu

**Affiliations:** 1 National Engineering Laboratory for AIDS Vaccine, School of Life Sciences, Jilin University, Changchun, P.R. China; 2 Center for New Medicine Research, Changchun University of Chinese Medicine, Changchun, P.R. China; 3 School of Pharmaceutical Sciences, Jilin University, Changchun, P.R. China; Helmholtz Zentrum Muenchen - German Research Center for Environmental Health, Germany

## Abstract

BST-2 blocks the particle release of various enveloped viruses including HIV-1, and this antiviral activity is dependent on the topological arrangement of its four structural domains. Several functions of the cytoplasmic tail (CT) of BST-2 have been previously discussed, but the exact role of this domain remains to be clearly defined. In this study, we investigated the impact of truncation and commonly-used tags addition into the CT region of human BST-2 on its intracellular trafficking and signaling as well as its anti-HIV-1 function. The CT-truncated BST-2 exhibited potent inhibition on Vpu-defective HIV-1 and even wild-type HIV-1. However, the N-terminal HA-tagged CT-truncated BST-2 retained little antiviral activity and dramatically differed from its original protein in the cell surface level and intracellular localization. Further, we showed that the replacement of the CT domain with a hydrophobic tag altered BST-2 function possibly by preventing its normal vesicular trafficking. Notably, we demonstrated that a positive charged motif “KRXK” in the conjunctive region between the cytotail and the transmembrane domain which is conserved in primate BST-2 is important for the protein trafficking and the antiviral function. These results suggest that although the CT of BST-2 is not essential for its antiviral activity, the composition of residues in this region may play important roles in its normal trafficking which subsequently affected its function. These observations provide additional implications for the structure-function model of BST-2.

## Introduction

The human immunodeficiency virus type 1 (HIV-1) exploits numerous positively acting cellular factors and pathways to maximize viral particle production [1]. However, mammalian cells also have multiple systems to suppress virus replication through the actions of innate host cell restriction factors [2]. Several host cell restriction factors have been identified that impact specific steps in the HIV-1 lifecycle. These are the APOBEC3 family of proteins [3], BST-2 [4], [5], [6], TRIM5α [7], [8], [9] and SAMHD1 [10], [11]. Viruses in turn have evolved to express adaptor molecules that antagonize these host cell restrictions, thereby allowing their replication to proceed efficiently. For example, HIV-1 Vif recruits cellular proteins to form an E3 complex to induce degradation of APOBEC3 proteins [12], [13]. The Vpu protein relieves the host restriction imposed by BST-2 [4], [5], and the Vpx protein induces proteasomal degradation of SAMHD1 [14], [15].

BST-2 is a 28- to 36-kDa type II single-pass transmembrane (TM) protein, and its expression varies between different cell types. It is constitutively expressed in Hela, H9, Jurkat and Molt4 cells, as well as primary T lymphocytes and macrophages, while it is absent in 293T, HOS and HT1080 cells [4]. However, BST-2 expression can be induced by type I interferon (IFN) to inhibit virus particle release from infected cells [4]. BST-2 exhibits broad antiviral activity against a wide range of enveloped viruses, such as HIV-1, HIV-2, simian immunodeficiency virus (SIV), other retroviruses, Lassa virus-like particles, and Marburg and Ebola virus-like particles (VLPs) [16], [17], [18]. During the late phase of the viral replication pathway, BST-2 causes nascent virions to remain trapped at the surface of the infected cell from which they are derived and to accumulate thereafter in endosomes following internalization [4], [5], [6]. BST-2 harbors an N-terminal cytoplasmic tail (CT), a single TM spanning region, an extracellular coiled-coil domain and a putative glycophosphatidylinositol (GPI) lipid anchor at its C-terminus [19]. Two potential N-linked glycosylation sites and three conserved cysteine residues are present in the extracellular domain [20]. An artificial tetherin-like protein, assembled from fragments of heterologous proteins, is able to mimic the biological activity of the native BST-2 [20]. Meanwhile, BST-2 proteins from other species such as pigs, cows and cats exhibit similar activities to that of human BST-2 [21], [22], [23]. Additionally, BST-2 was identified as an inducer of nuclear factor-kappa B (NF-κB) activation in a whole-genome transfection screen [24]. Critical NF-κB signaling residues, mainly located in the cytoplasmic and ectodomain, include Y6, Y8, RVP (10–12), three cysteines (53, 63, 91), two asparagines (65, 92), L70 and L123 [25], [26], [27]. Moreover, the effect of the deletion of the GPI anchor is under debate [25], [27]. The Vpu protein is the viral antagonist of BST-2 encoded by HIV-1 [4], [5]. It is a 16 kDa type I TM protein comprised of a short N-terminal region, a TM domain and a longer cytoplasmic domain (CD) [28], [29], [30], [31]. The precise mechanism of how Vpu antagonizes BST-2 is still in debate. However, there is general agreement that Vpu co-localizes with BST-2 and reduces the cell surface level of BST-2, either by retarding its delivery to the plasma membrane or by enhancing its internalization [32], [33]. Meanwhile, Vpu also degrades the total cellular level of steady-state BST-2 [34], [35]. The expression of HIV-1 Vpu inhibits the activation of NF-κB by BST-2, and this inhibition requires the ability of Vpu to bind the cellular β-TrCP-E3-ubiquitin ligase complex [27]. The SIV Nef protein and other lentiviral envelope proteins are also known to antagonize BST-2 [36], [37], [38], [39], [40].

Although relationships between the structural domains and the biological functions of BST-2 have been constantly delineated since its first discovery, uncertainties remain. For example, some research groups have highlighted several distinct functions of the CT domain of human BST-2. The deletion of each structural domain on the N-terminal HA-tagged BST-2 was initially reported to result in severe impairment of its anti-HIV-1 virion release function [4]. Tokarev *et al.* declared that a few specific serine/threonine and lysine residues in the CT are potential sites for Vpu-induced ubiquitination of BST-2 [41]. However, another group has proposed a different view indicating that the ubiquitination of BST-2 by Vpu does not require lysine, serine or threonine residues within the BST-2 CT [42]. Moreover, Miyakawa *et al.* reported a BST-2-associated protein BCA2/Rabring7 acting as a co-factor for BST-2 and localized the site of its interaction to the CT of BST-2 [43]. In addition, the cysteines in the CT potentially contribute to the homo-dimerization of BST-2 [44]. However, recent studies indicated that feline BST-2 has a very short N-terminal cytoplasmic region compared with those of other mammalian and non-mammalian homologs. Surprisingly, it significantly blocks budding of FIV, HIV-1, HIV-2 ROD10, SIVmac239 and RD-114 particles [23], [40]. An artificial feline BST-2 mutant containing a longer N-terminal cytoplasmic homolog only exhibits limited antiviral capability [40]. Moreover, ovine BST-2A, one of the two ovine BST-2 isoforms, also retains a CT shorter than that of human BST-2. Interestingly, this shorter isoform displays a stronger antiviral activity than the longer isoform [21]. Cocka and Bates showed in a recent paper that naturally occurring variants of human BST-2 initiating from the internal ATG are partially resistant to Vpu [26]. Based on these observations, we hypothesized that the long N-terminal cytoplasmic region of the BST-2 protein may confer an unnecessary role to its antiviral function in some mammals and non-mammals.

In this study, we explored the impact of mutations in the CT of BST-2 on its antiviral function against HIV-1. The BST-2 protein with a truncated CT still exhibited potent antiviral activity against Vpu-defective HIV-1, which put into question the functional necessity of this domain. In addition, the truncated BST-2 also displayed relatively stronger inhibitory effects against Vpu-positive HIV-1 than the wild-type BST-2 protein. Interestingly, the fusion of different epitope labels to the N-terminus of the CT-truncated BST-2 impaired its antiviral activity to varying degrees. In particular, an N-terminal HA-tagged CT-truncated BST-2 retained little antiviral activity. Notably, its intracellular localization and N-glycosylation state changed dramatically, along with the lower cell surface expression. These observations suggest that the CT of BST-2 may be unnecessary for its antiviral activity, although the specific N-terminal sequence close to the TM domain could impair BST-2 trafficking. However, the CT may further contribute to Vpu sensitivity of BST-2 in addition to the TM domain interaction. Additionally, the CT-truncated BST-2 and N-terminal or internal HA-tagged BST-2 partially lost the ability to induce NF-κB activation. These results provide additional implications for the structure-function model of BST-2 and its interaction with HIV-1 Vpu. The results also indicate that certain modifications of BST-2 may affect one biological function while not affecting another function.

## Materials and Methods

### Cell cultures and transfections

HEK293T cells (ATCC, No. CRL-11268) and COS-7 cells (ATCC, No. CRL-1651) were maintained in Dulbecco’s high glucose modified Eagle’s medium (DMEM) supplemented with 10% heat-inactivated fetal bovine serum (FBS), 0.29 mg/ml L-glutamine, and 100 units/ml penicillin/streptomycin. Plasmid transfections in 293T cells and COS-7 cells were performed using Lipofectamine 2000 according to the manufacturer’s instructions (Invitrogen, Carlsbad, CA, USA).

### Plasmids

All of the modified BST-2 proteins and mutants, including BST-2 NHA, BST-2 IHA, BST-2 ΔN20 NHA, BST-2 ΔN20 IHA, BST-2 ΔN20 NT (no tag), BST-2 ΔN20 NMyc, BST-2 ΔN20 NFlag, BST-2 ΔN20 NHARD, BST-2 ΔTM 159H6 (with a 6×His tag inserted in-frame at residue 159 followed by a stop codon), BST-2 ΔN20 IHA 159H6 and BST-2 ΔN20 NHA 159H6, BST-2 ΔKRK (K18A, R19A, K21A), BST-2 ΔN12 NT, BST-2 ΔN12 NHA were engineered based on BST-2 WT NT (no tag) with the use of the QuickChange mutagenesis system (Santa Clara, CA, USA), and sequences were confirmed. All primers were synthesized by the solid phase phosphoramidite triester method (ShineGene, Shanghai, China). The HIV-1 proviral clone pNL4-3, HIV-1 Vpu-defective pNL4-3 ΔVpu, BST-2 expression plasmid (BST-2 WT NT), pEGFP-N3 and VR1012 have been described previously [45], [46], [47].

### Antibodies

The following antibodies were used in this study: anti-HA mouse mAb (Covance, Emeryville, CA, USA), anti-myc mouse mAb (Millipore, Billerica, MA, USA), anti-tubulin mouse mAb (Covance, Princeton, NJ, USA), anti-His mouse mAb (Sigma, St. Louis, MO, USA), anti-Flag mouse mAb (Sigma), anti-BST-2 mouse mAb (Abnova, Taipei, Taiwan), anti-BST-2 rabbit polyclonal antibody (pAb) obtained from the National Institutes of Health, AIDS Research and Reference Reagent Program (NIH-ARRRP, Germantown, MD, USA), and anti-p24 mouse mAb obtained from an HIV-1 p24 hybridoma (NIH-ARRRP).

Alkaline phosphatase conjugated goat anti-rabbit or anti-mouse IgG secondary antibodies were purchased from Jackson Immunoresearch Laboratories (West Grove, PA, USA). Alexa Fluro 488 or 633 goat anti-mouse IgG secondary antibodies were from Invitrogen.

### Western blotting

Cells were collected 48 h after transfection. Cell lysates were prepared by washing cells in PBS and lysing in RIPA buffer (150 mM NaCl, 50 mM Tris, 1% Triton X-100, 0.1% SDS) for 30 min at 4°C, followed by addition of 4× SDS sample buffer (1 M Tris, pH 6.8, with 8% SDS, 40% glycerol, 0.4 M dithiothreitol, and 0.8% bromophenol blue). The samples were boiled for 10 min, and proteins were separated by SDS-PAGE. The proteins were transferred onto nitrocellulose membranes by semi-dry transfer (Bio-Rad, Hercules, CA, USA). After blocking in 5% non-fat milk, the membranes were probed with various primary antibodies against proteins of interest. Secondary antibodies were alkaline phosphatase-conjugated anti-rabbit, anti-mouse IgG (Jackson Immunoresearch), and staining was carried out with 5-bromo-4-chloro-3indolyl phosphate (BCIP) and nitro blue tetrazolium (NBT) solutions.

### Immunofluorescence analysis

Evaluation of cell-associated BST-2 protein by immunofluorescence was performed as follows. COS-7 cells (20–50% confluent) seeded on coverslips in a 24-well plate were transfected with plasmid DNA using Lipofectamine 2000. At 48 h post-transfection, cells were fixed with 4% paraformaldehyde in PBS for 10 min at room temperature. For intracellular immunofluorescence, experiments were performed by permeabilizing the cells in 0.1% Triton X-100 for 8 min. Then, the cells were washed three times in PBS, blocked in 10% FBS in PBS and then incubated with a mouse anti-BST-2 mAb diluted 1∶1000 in PBS containing 1% FBS for 1 h at room temperature. Cells were washed three times in PBS and stained with Alexa Fluor 488 goat anti-mouse IgG (Invitrogen) diluted 1∶1000 in PBS (1% FBS) along with 1 mg/ml DAPI (4,6-diamidino-2-phenylindole) for 1 h at room temperature. Subsequently, the cells were washed three times in PBS and stained ER for 15 min at 37°C with ER-Tracker Red (Molecular Probes, Invitrogen), which is a BODIPY TR-conjugated glibenclamide, a low-molecular weight drug specially binds sulphonylurea receptor (SUR) on the ER. For surface immunofluorescence analysis, the same protocol was followed, except the Triton X-100 step was not performed. Stained cells were washed three times in PBS, and then fluorescent images were obtained using a Zeiss LSM710 confocal microscopy equipped with a 40× objective.

### Flow cytometric analysis

293T cells plated in 6-well plates were transfected with indicated expression plasmids along with the pEGFP-N3 vector (Clontech, Mountain View, CA, USA) expressing EGFP as a marker of transfection efficiency. After 48 h, cells were fixed with 4% paraformaldehyde in PBS for 40 min at room temperature. After fixation, cells were first blocked for 10 min in 10% FBS in PBS. Thereafter, BST-2 was stained with an anti-BST-2 mAb, followed by Alexa Fluor 633 conjugated goat anti-mouse IgG (Molecular Probes, Invitrogen) and analyzed on a MoFlo XDP cell sorter (Beckman Coulter, Brea, CA, USA).

### Virion production and infectivity assay

HIV-1 particles were produced by transient transfection of HeLa or 293T cells in a 6-well plate with a proviral construct and indicated amount of other plasmids. Two days later, supernatants (2 ml per culture) from producer cells were harvested, clarified by centrifugation and passed through a 0.22 m filter. The viral particles were pelleted through a 20% sucrose layer at 110,000×*g* for 1 h and resuspended in 30 µl RIPA buffer. Virus particle pellets and corresponding cell lysates were analyzed by SDS-PAGE and Western blot using an anti-p24 capsid antibody. In single-cycle infectivity assays, 50 µl of the filtered supernatant was mixed with DEAE-dextran (Sigma) at a final concentration of 15 µg/ml and incubated with TZM-bl indicator cells in a 96-well plate. At 48 h post-infection, the cells were lysed, mixed with luciferase substrates and assayed for luciferase activity using a fluorescence microplate reader to represent released virion yield.

### Protein purification and N-terminal sequencing

Approximately 48 h after transfection, cells were harvested in PBS and centrifuged at 500×*g* for 10 min. The cell pellet was resuspended in 2 ml cell lysis buffer containing 150 mM NaCl, 50 mM Tris-Cl, 0.5% Triton X-100, 5% glycerol and protease inhibitor (Roche, Basel, Switzerland). The cell suspension was sonicated for 10 min on ice, and then centrifuged at 16,000×*g* for 10 min. The supernatant was applied to a column containing 1 ml ProBond nickel-chelating resin (Invitrogen). The flow-through fractions were collected, and the column was washed with three bed volumes of wash buffer containing 150 mM NaCl, 50 mM Tris-Cl, 5% glycerol and an increasing gradient of imidazole (10 mM, 20 mM, 35 mM and 50 mM final concentration). The proteins were eluted with two bed volumes of wash buffer containing 500 mM imidazole and concentrated to the final volume of 500 µl. The proteins were mixed with SDS sample buffer, separated by SDS-PAGE and then transferred onto polyvinylidene fluoride (PVDF) membranes by semi-dry transfer and 1× CAPS buffer (10 mM CAPS, 20% methanol, pH 11.0). The Edman degradation method was used for N-terminal sequencing, which was provided by the Protein Sequencing Room, College of Life Science, Peking University.

### PNGase F digestion of BST-2

293T cells transfected with wild-type or BST-2 mutants were lysed using 1% NP40 in PBS on ice for 5 min, and nuclei were removed by centrifugation. Lysates were heated at 100°C for 10 min to denature the proteins. The reaction was set up in a total volume of 20 µl by adding 2 µl 10×G7 reaction buffer and 2 µl 10% NP40 to 16 µl lysate and incubated with or without 1000 U of PNGase F (New England Biolabs) at 37°C 2 h prior to Western blot analysis with an anti-BST-2 pAb.

### Cellular fractionation

293T cells plated in 10-cm dishes were transfected with the plasmid of indicated BST-2 variants. After 48 hours incubation, the cells were mixed with 1% Triton X-100 PBS buffer, rocking on ice gently. The whole cell lysate was brought up to a sucrose gradient for isolation. The sucrose layer was prepared in a 12 ml centrifuge tube composed by each of 1 ml 20%, 30%, 40%, 50%, 60% sucrose in PBS. The gradients were spun at 35,000 rpm in a Beckman SW41 swing out rotor for 16 hours at 4°C. Eleven 0.5-ml fractions including the upper sample were collected from the top of the gradient.

### Luciferase assay for detecting NF-κB activity

293T cells plated in 24-well plates were transfected with 25 ng BST-2 expression plasmids along with the 250 ng pNF-κB-Luc reporter plasmid (Agilent) and 50 ng Renilla luciferase plasmid as a transfection marker. Two days later, cell lysates were harvested in Triton X-100 lysis buffer and transferred to a black flat bottom 96-well plate. Luciferase Assay System substrate and Stop-GLO buffer (Promega) was added to the lysates according to the manufacturers’ instructions. Samples were analyzed in a Multilabel Plate Reader (Perkinelmer, VICTOR X2). The raw luciferase value was normalized to the renilla luciferase value and compared with the mock control.

### Sequence analysis and accession numbers

DNA sequences were aligned by ClustalX and edited by hand based on amino acid sequence. The sequences of the twenty primate BST-2 genes used in the sequence alignment are referred to a previous study [48] and recorded in the GenBank database under the accession numbers NM_004335, GQ864267, and HM136905-HM136922.

## Results

### Cloning and characterization of BST-2 variants

**Figure 1:**
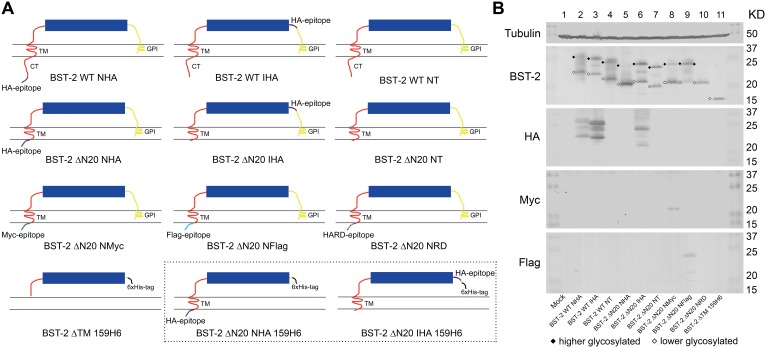
Schematic representation and basic characteristics of BST-2 variants. (A) Deletion mutagenesis of BST-2 N-terminus. The CT domain of BST-2 (red curved line under the cell membrane), fused HA peptide (purple), Myc peptide (green), Flag (cyan) and 6×His peptide (black) are represented. The constructs in the dashed box are specially designed for the nickel affinity purification and which expression test were shown in Figure 3. (B) SDS-PAGE analysis of BST-2 variants under denaturing conditions. 293T cells were transfected with 500 ng of VR1012, BST-2 WT NHA, BST-2 WT IHA, BST-2 WT NT, BST-2 ΔN20 NHA, BST-2 ΔN20 IHA, BST-2 ΔN20 NT, BST-2 ΔN20 NMyc, BST-2 ΔN20 NFlag or BST-2 ΔTM 159H6. After 48 h, cells were harvested and analyzed by Western blotting using an anti-BST-2 pAb, anti-HA mAb, anti-myc mAb, anti-flag mAb and anti-tubulin mAb, respectively. The major bands corresponding to higher and lower glycosylated BST-2 are marked with symbols.

To better understand the functional significance of the N-terminal cytoplasmic region of BST-2, we studied the impact of its truncation on the antiviral function and other characteristics of this protein. First, we constructed a CT-truncated form of BST-2, designated as BST-2 ΔN20. Commonly, the detection of BST-2 mostly relies on the fusion of the epitope tag at the N-terminus or interior sequence. As shown in Figure 1A, in order to determine the possible influence of an HA tag on the wild-type or CT-truncated BST-2, it was either added to the N-terminus of the protein or internally in the ectodomain close to the GPI anchor as tested in a previous report [20]. According to our preliminary data of N-terminal HA tagged CT-truncated BST-2, either Myc, Flag or HA random (RD) tags was then fused to the N-terminus of BST-2 ΔN20 to test the different effects. BST-2 ΔTM 159H6, a mutant lacking both membrane spanning domains was set as a control. The constructs with 6His tags in the dashed box are designed for the nickel affinity purification.

Lysates of 293T cells transfected with expression plasmids for wild-type or mutant BST-2 were separated by SDS-PAGE and analyzed by Western blot to determine the basic properties of the variants. The patterns of most mutants were similar to that of the wild-type, the higher and lower glycosylated forms were marked. BST-2 ΔN20 NHA presented as only a single band corresponding to a lower molecular weight protein (Fig. 1B). BST-2 ΔN20 NRD exhibited a very similar profile while BST-2 ΔN20 NMyc to a less extent. Interestingly, most constructs can be detected with antibodies corresponding to their epitope tags, except for BST-2 ΔN20 NHA, which was hardly detected by the HA antibody. With about 110 residues, BST-2 ΔTM 159H6 exhibited as a single band corresponding to a lower molecular weight protein around 15 KDa.

### N-terminally deleted BST-2 inhibits Vpu-defective and wild-type HIV-1 particle release

**Figure 2:**
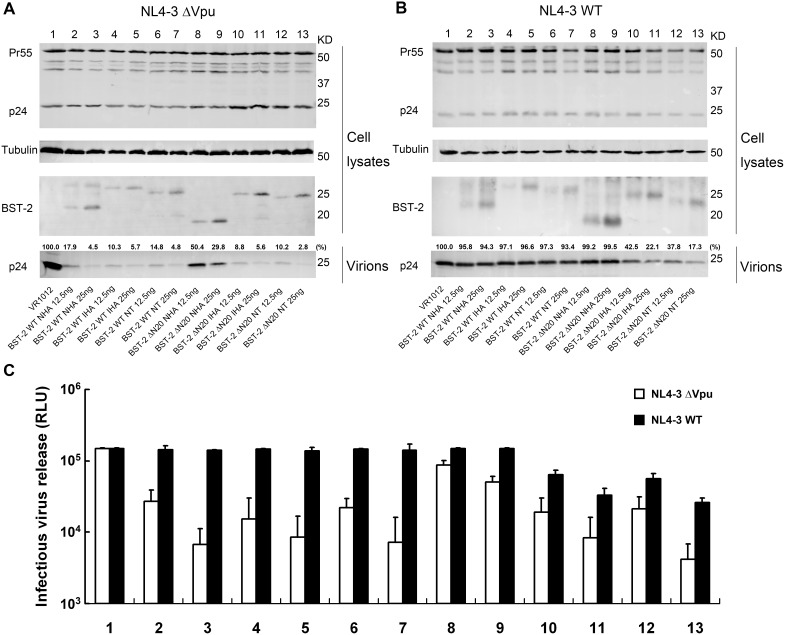
Different BST-2 mutants show different inhibitory effects on Vpu-defective or wild-type virus particle release. (A and B) BST-2 WT NHA (12.5 ng or 25 ng), BST-2 WT IHA (12.5 ng or 25 ng), BST-2 WT NT (12.5 ng or 25 ng), BST-2 ΔN20 NHA (12.5 ng or 25 ng), BST-2 ΔN20 IHA (12.5 ng or 25 ng), BST-2 ΔN20 NT (12.5 ng or 25 ng) or the VR1012 control vector (adjusted to 25 ng) was co-transfected with 1 µg of the proviral plasmid pNL4-3 ΔVpu or pNL4-3 WT in 293T cells. At 48 h post-transfection, cultured supernatants were ultracentrifuged to concentrate the virus particles. Virions and cell lysates were analyzed by Western blotting using an anti-p24 antibody to detect viral p24 and intracellular Pr55Gag proteins, an anti-BST-2 pAb to detect BST-2 and an antibody against tubulin to assess sample loading. The blots of released p24CA were quantified using Bandscan software and normalized by tubulin levels. (C) The titration of the released infectious viruses in A and B are shown in columns below the Western blotting, which are representative of three independent experiments.

The CT-truncated BST-2 protein (BST-2 ΔN20) with an internal or N-terminal HA tag was initially analyzed to determine if it still retained antiviral activity. 293T cells were co-transfected with BST-2 WT NHA, BST-2 WT IHA, BST-2 WT NT, BST-2 ΔN20 NHA, BST-2 ΔN20 IHA, BST-2 ΔN20 NT or VR1012 and pNL4-3 ΔVpu. The cell lysates and released capsid proteins were then analyzed by Western blotting. To make the comparison between the BST-2 variants more accurate, the released infectious virions were further measuring by titrating the cultured supernatants shown below. The Vpu-defective HIV-1 viral particles exhibited significant impairment in release from cells expressing wild-type BST-2 (Fig. 2A, lane 2–7), while BST-2 ΔN20 IHA and BST-2 ΔN20 NT also showed considerable antiviral activity, which was enhanced with increasing doses (Fig. 2A, lanes 10–13). Importantly, BST-2 ΔN20 NHA only showed limited antiviral activity (Fig. 2A, lane 8 and 9). The above results indicated that the N-terminal HA tag negatively impacted the antiviral function of BST-2 with a truncated CT.

The regions of interaction between human BST-2 and its viral antagonist HIV-1 Vpu have been localized to their hydrophobic TM domains [32]. To evaluate if alterations in the BST-2 cytoplasmic region adjacent to the TM domain would have any impact on the interaction of the two proteins, we detected the sensitivity of these BST-2 variants to HIV-1 Vpu by evaluating their abilities to inhibit wild-type HIV-1. 293T cells were co-transfected with BST-2 WT NHA, BST-2 WT IHA, BST-2 WT NT, BST-2 ΔN20 NHA, BST-2 ΔN20 IHA, BST-2 ΔN20 NT or VR1012 and pNL4-3, followed by analysis of the cell lysates and released capsid proteins by Western blotting. The Vpu-positive HIV-1 could antagonize the host restriction and released normally in the presence of wild-type BST-2 (Fig. 2B, lanes 2–7). Significantly, the functional mutants with a shortened N-terminus, BST-2 ΔN20 IHA and BST-2 ΔN20 NT, inhibited the Vpu-encoding HIV-1, which was enhanced with increasing doses, and were even somewhat more effective than the wild-type BST-2 (Fig. 2B, lanes 10–13). To strengthen the P24 capsid release results and further demonstrate the comparison between the virus samples, we measured infectious virions release by titrating the clarified cultured supernatants on TZM-bl cells and shown in columns (Fig. 2C). The above results demonstrated that the BST-2 ΔN20 NHA mutant was non-functional and exhibited no antiviral activity (Fig. 2B, lanes 8 and 9).

Similar experiments were performed to analyze the short form BST-2 (BST-2 ΔN12) and BST-2 ΔN20. As shown in Fig. S1A, BST-2 ΔN12 also exhibited considerable activities against the virus release of HIV-1 ΔVpu. While the fusion of the NHA tag also partially affected the antiviral function of BST-2 ΔN12 (Fig. S1B, C).

### Altered apparent molecular size of BST-2 ΔN20 NHA is not due to proteolytic cleavage of the epitope-tagged N-terminus

Aside from the dysfunction of BST-2 ΔN20 NHA as mentioned above, this mutant migrated as a single species in the SDS-PAGE gel, which was smaller than any of the BST-2 ΔN20 IHA bands. Two possible explanations may account for the altered size of the protein in SDS-PAGE analysis: digestion at specific proteolytic sites by cellular enzymes and the absence of post-translational modification. We initially hypothesized that the 20-amino acid deletion of BST-2 and the fusion of an N-terminal HA tag may generate a secretory leader peptide sequence, which can be cleaved by cellular endogenous protease during expression. The result would be an incomplete protein devoid of most of the N-terminus, similar to the generation of a BST-2 delTM mutant in a previous study [20].

**Figure 3:**
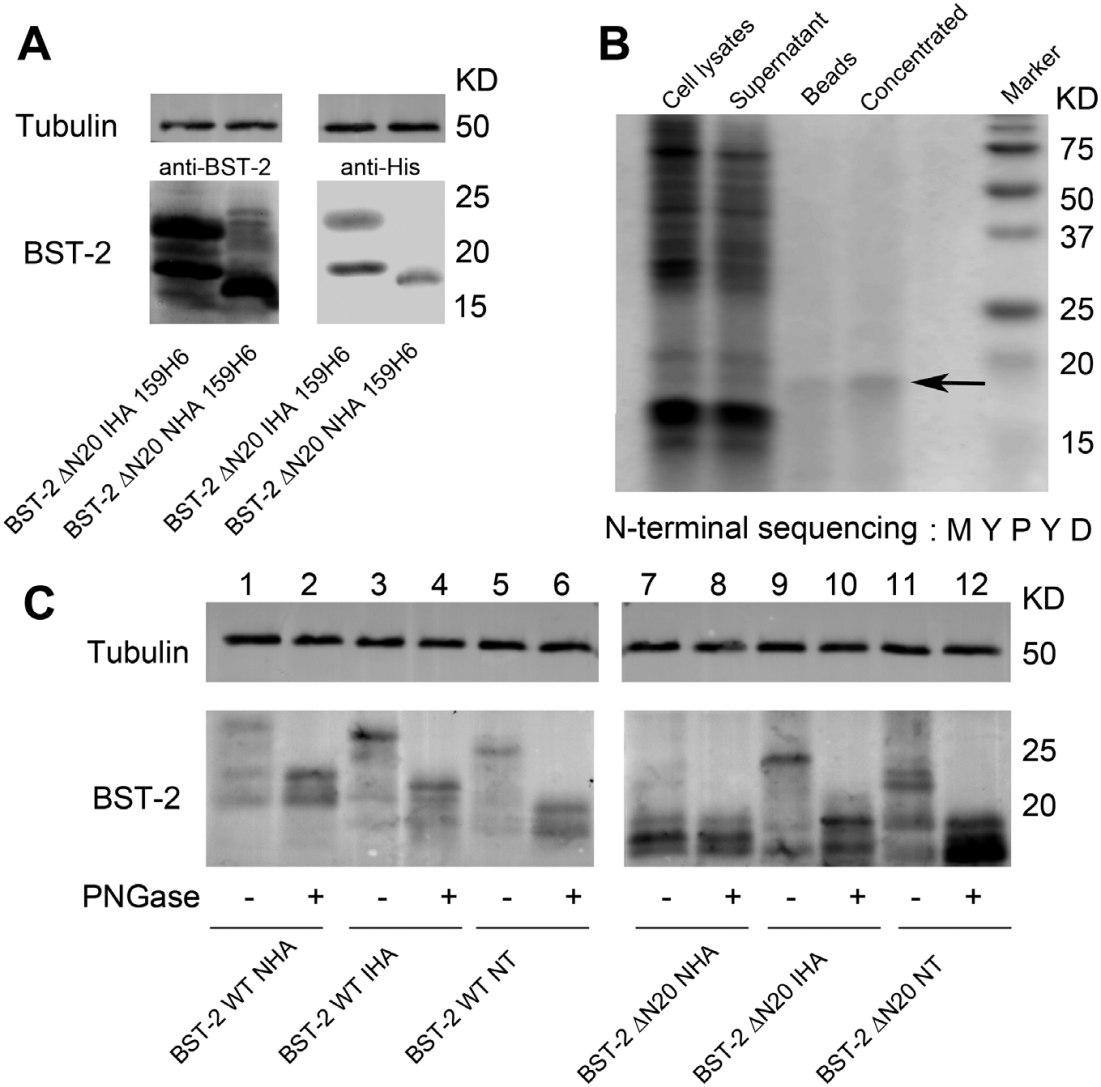
N-terminal sequencing of BST-2 ΔN20 NHA and glycosylation analysis. (A) 293T cells were co-transfected with 500 ng of BST-2 ΔN20 IHA 159H6 or BST-2 ΔN20 NHA 159H6. After 48 h, cells were harvested and analyzed by Western blotting using an anti-BST-2 pAb, anti-His mAb and anti-tubulin mAb. (B) 293T cells in two T125 flasks were transfected with 6 µg of BST-2 ΔN20 NHA 159H6 per flask. Cells were harvested 48 h after transfection, lysed and separated by nickel affinity chromatography. Samples collected from the purification procedures were separated by SDS-PAGE with Coomassie Brilliant Blue staining and the concentrated BST-2 protein was labeled with an arrow. N-terminal sequencing results are shown below. (C) BST-2 WT or variant expression plasmids (500 ng) were transfected into 293T cells. Cells were harvested 48 h after transfection and divided into two portions. Cell lysates were incubated in the presence or absence of PNGase F and analyzed by Western blotting using an anti-BST-2 pAb and anti-tubulin mAb. This experiment was repeated for three times and the most representative data was shown.

To test the hypothesis above, the N-terminal sequence was removed from this mutated protein. The dual membrane associated region of BST-2 increases the difficulty in producing its full-length protein in a soluble and properly folded form. Therefore, crystal structure studies of BST-2 have been limited only to its ectodomain [49], [50], [51], [52]. However, removal of the putative GPI-anchored modification signal domain in BST-2 has been shown to increase soluble BST-2 protein levels in the lysis buffer [4], [20]. To increase the solubility of the protein for efficient affinity purification while maintaining as much of the N-terminus as much as possible for sequencing, we constructed two plasmids expressing BST-2 ΔN20 NHA and BST-2 ΔN20 IHA with a 6×His tag inserted in-frame at residue 159 followed by a stop codon (abbreviated as 159H6) [53]. 293T cells were transfected with either of the two 6×His tag mutants and then analyzed by SDS-PAGE and Western blotting with the corresponding antibody. From the analysis of the electrophoretic patterns of these double-tagged mutants, we could firmly rule out that the removal of the GPI anchor had any influence on the aforementioned phenomenon caused by different N-terminal sequences (Fig. 3A). After a non-denaturing cell lysing procedure and affinity purification with nickel-chelating beads, the BST-2 ΔN20 NHA 159H6 protein was separated by PAGE. N-terminal sequencing of this protein yielded the sequence N-MYPYD (Fig. 3B), which was perfectly matched with the HA tag. With these results, we confirmed that the BST-2 ΔN20 NHA protein still maintained all of its N-terminus and was not cleaved by an endogenous proteolytic enzyme.

### Glycosylation of BST-2 ΔN20 NHA is impaired even though it contains all putative N-glycosylated sites

BST-2 migrates as a number of species around 28- to 36-kDa upon SDS-PAGE analysis, presumably due to the heterogeneity in post-translational modifications, especially N-linked glycosylation. A previous study showed that mutation at both of the putative N-linked glycosylation sites (N65A and N92A) reduced the apparent molecular weight of both transiently and stably expressed tetherin to 21 kDa. Meanwhile, treatment of the stably expressed 32–38 kDa BST-2 protein with peptide-N-glycosidase-F (PNGase F) reduced its molecular weight to a similar degree, as did mutation of both glycosylation sites [20]. Although human BST-2 migrated primarily as a 28 kDa species when transiently overexpressed in 293T cells because of an insufficient rate of addition of complex carbohydrates, either mutation of N-glycosylated sites or PNGase F was also capable of reducing the apparent molecular size of the protein band. In addition, the N65, 92A double mutant was almost completely inactive. The abovementioned non-functional BST-2 ΔN20 NHA mutant coincidentally was similar to the BST-2 mutant carrying double glycosylated site mutations in that it appeared as only one small band, potentially due to impairment of glycosylation. After transfection of 293T cells, the cell lysates containing BST-2 WT or each variant were treated with PNGase F. As shown in Fig. 3C, each PNGase F-treated protein showed a reduced molecular weight, except for the BST-2 ΔN20 NHA mutant, which was nearly the same size as that before enzymatic treatment. This result suggested that the normal glycosylation process of BST-2 ΔN20 NHA was impaired, although it still contained all putative N-glycosylated sites.

### Aberrant cellular localization of BST-2 ΔN20 NHA

**Figure 4:**
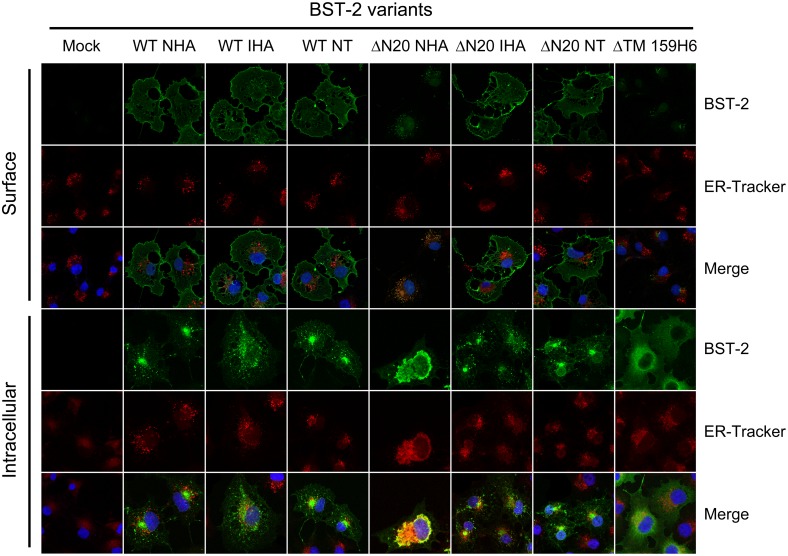
Intracellular localization of BST-2 variants. COS-7 cells transfected with 200 ng of control plasmid VR1012, BST-2 variants expression plasmids were observed by confocal microscopy with surface staining and intracellular staining; blue, cell nucleus; green, BST-2 protein; red, ER. Images were taken under a Zeiss LZM710 confocal microscope. At least 30 independent cells were examined in each sample, and the most representative cells are shown.

BST-2 is constitutively recycled between the plasma membrane, endosomal and trans-Golgi network (TGN) compartments [54], although it localizes mainly to the plasma membrane and exhibits a puncta-like distribution [4]. In the current study, subcellular distributions of BST-2 variants were detected to verify if the alteration of glycosylation was associated with changes in cellular localization. In this assay, a presumptive negative control mutant BST-2 ΔTM 159H6 was introduced, with a deletion of the N-terminus including the TM domain and GPI anchor, which may lead to the total elimination of the interaction between BST-2 and the lipid membrane. Subconfluent monolayers of COS-7 cells were transfected with empty vector, BST-2 WT NHA, BST-2 WT IHA, BST-2 WT NT, BST-2 ΔN20 NHA, BST-2 ΔN20 IHA, BST-2 ΔN20 NT, BST-2 ΔTM 159H6. Surface and intracellular localization was evaluated by an indirect confocal immunofluorescence technique using an anti-BST-2 monoclonal antibody (mAb). As shown in Fig. 4, the surface distributions of BST-2 WT NHA, BST-2 WT IHA, BST-2 ΔN20 IHA, BST-2 ΔN20 NT were similar as with BST-2 WT NT. In contrast, BST-2 ΔN20 NHA and BST-2 ΔTM 159H6 showed little surface distribution and also partially lost the intracellular puncta-like pattern, especially the putative negative control BST-2 ΔTM 159H6. Furthermore, the intracellular distribution of BST-2 ΔTM 159H6 was relatively diffuse throughout the cell, which was very similar to that of some cytosolic proteins such as green fluorescent protein (GFP). However, unlike this control protein, BST-2 ΔN20 NHA appeared as perinuclear clusters around the endoplasmic reticulum (ER), and this pattern is quite different from the punctate-like trafficking compartments of BST-2 WT. Additionally, with the analysis of the original images containing more cells, we noticed that the ER membranes also appear exaggerated in the presence of BST-2 ΔN20 NHA but not BST-2 ΔN20 NT (Fig. S2).

### Cell surface expression of BST-2 ΔN20 NHA is markedly reduced

**Figure 5:**
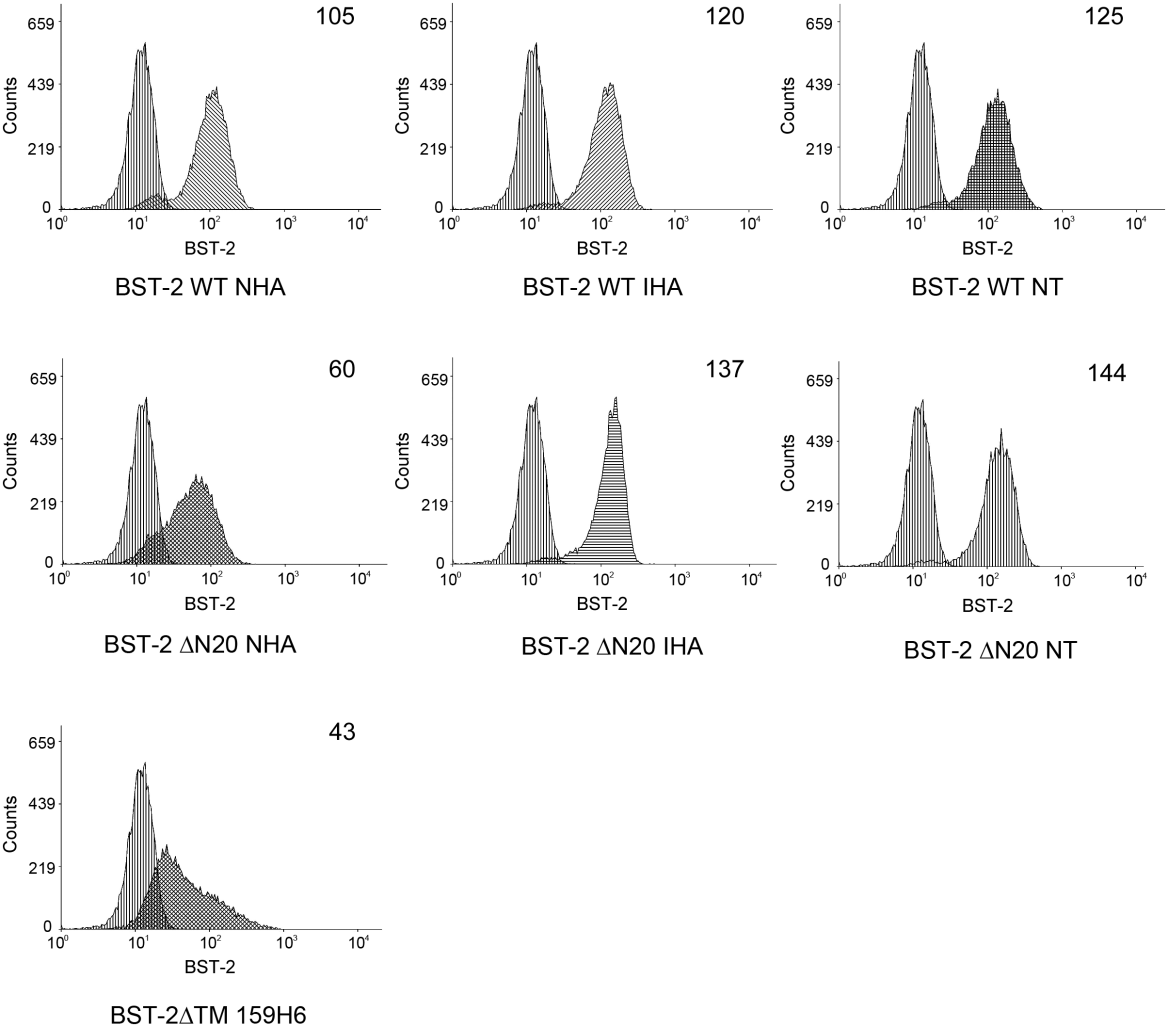
Cell surface expression of BST-2 WT and ΔN20 variants. 293T cells were co-transfected with 500 ng of VR1012, BST-2 WT or **ΔN20** variant expression plasmids, along with 500 ng of pEGFP-N3 as a transfection marker. After 48 h, cells were harvested and stained with the anti-BST-2 mAb and Alexa 633 goat anti-mouse IgG, followed by flow cytometric analysis. Cells only transfected with pEGFP-N3 was used as a negative control. The samples were gated on EGFP+ cells, and the surface BST-2 levels are shown in histograms with mean fluorescent intensitiy values at the top right corner. This experiment was repeated for three times and the most representative data was shown.

The exact mechanism for BST-2-mediated restriction of HIV-1 viral particle release is still not fully defined, although most researchers accept that BST-2 must appear at the cell surface to exert its antiviral function. In order to investigate the effect on cell surface localization of BST-2 by changing its N-terminal cytoplasmic region, the following FACS analysis was carried out. BST-2 has both a TM-spanning domain and a GPI lipid anchor, which are largely responsible for its membrane association. The EGFP-encoding vector pEGFP-N3, used to verify the transfection efficiency for FACS analysis, was co-transfected in 293T cells with each BST-2 variant (BST-2 WT NHA, BST-2 WT IHA, BST-2 WT NT, BST-2 ΔN20 NHA, BST-2 ΔN20 IHA, BST-2 ΔN20 NT, or BST-2 ΔTM 159H6). By flow cytometry, levels of cell surface expression of these proteins were evaluated with a BST-2 mAb and Alexa-633-conjugated anti-mouse secondary antibody. Cells transfected with only pEGFP-N3 were used as negative controls. Samples were gated on EGFP+ cells, and the surface BST-2 levels are shown in histograms with mean values (Fig. 5). Even with a CT truncation, BST-2 ΔN20 IHA and BST-2 ΔN20 NT on the cell membrane were detected at fundamentally normal levels compared with the wild-type BST-2. However, the BST-2 surface level of BST-2 ΔN20 NHA expressing cells decreased remarkably, which is dramatically lower than BST-2 WT expressing cells. Moreover, the control mutant BST-2 ΔTM 159H6 exhibited a severely impaired surface distribution. Similar experiments were performed to analyze the short form BST-2 (BST-2 ΔN12). As shown in Fig. S1D, BST-2 ΔN12 NHA exhibited moderately condensed distribution in perinuclear region which is similar with BST-2 ΔN20 NHA, but BST-2 ΔN12 NT show no such tendency.

### The antiviral function of CT-truncated BST-2 is affected by different epitoped tags

The above experimental results revealed that although BST-2 ΔN20 IHA, BST-2 ΔN20 NT and BST-2 ΔN20 NHA lacked the N-terminal 20 residues extending into the cytosol, their antiviral functions and post-translational modifications along with the cellular distribution profiles were markedly distinct. All these observations led to the prediction that the abnormal behavior of BST-2 ΔN20 NHA was related to the additional N-terminal HA tag. Whether this was a specific consequence of a gain of function from the HA tag or just a result of the addition of a redundant short peptide required clarification. Therefore, we designed a number of BST-2 ΔN20 mutants with distinct N-terminal tags, including a Myc tag, Flag tag and N-terminal random (NHARD) sequence based on the HA tag. In the SDS-PAGE analysis, BST-2 ΔN20 NHARD migrated as a single species similar to BST-2 ΔN20 NHA (Fig. 1B lane 10). This phenomenon was consistent with the non-glycosylated profile of BST-2 ΔN20 NHA. The amount of glycosylated BST-2 ΔN20 NFlag expressed seemed to be greater than that of BST-2 ΔN20 NMyc (Fig. 1B lane 8, 9).

**Figure 6:**
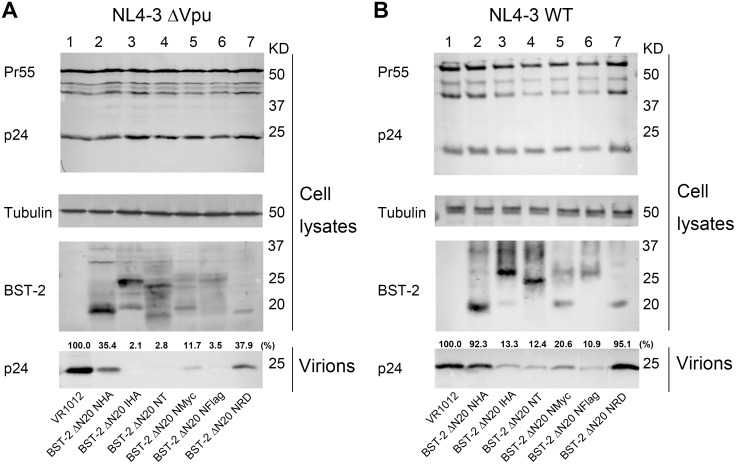
Fusion of different epitope tags to N-terminus of BST-2 ΔN20 results in different effects on antiviral function. (A and B) BST-2 ΔN20 IHA, BST-2 ΔN20 NHA, BST-2 ΔN20 NT, BST-2 ΔN20 NMyc, BST-2 ΔN20 NFlag, BST-2 ΔN20 NHARD or control vector VR1012 (25 ng each) was co-transfected with 1 µg of the proviral plasmid pNL4-3 ΔVpu or pNL4-3 WT in 293T cells. At 48 h post-transfection, cultured supernatants were ultracentrifuged to concentrate the virus particles. Virions and cell lysates were analyzed by Western blotting. This experiment was repeated for three times and the most representative data was shown.

Next, antiviral functions of these BST-2 variants (BST-2 ΔN20 NHA, BST-2 ΔN20 IHA, BST-2 ΔN20 NT, BST-2 ΔN20 NMyc, BST-2 ΔN20 NFlag, BST-2 ΔN20 NHARD) or VR1012 as a negative control were determined by co-transfection with a Vpu-defective HIV-1 proviral construct (pNL4-3 ΔVpu) in 293T cells. The cell lysates and released capsid proteins were analyzed subsequently by Western blotting. Consistent with the results above, the antiviral activity of BST-2 ΔN20 NHA was severely impaired (Fig. 6A, lane 2), while the activity of the mutant with a random NHA tag (NHARD) showed almost no difference compared with that of the original NHA (Fig. 6A, lane 7). The two BST-2 ΔN20 mutants with an NMyc or NFlag tag both exhibited considerable antiviral activity, while BST-2 ΔN20 NFlag showed a slightly greater activity level than that of BST-2 ΔN20 NMyc. The activities of all these proteins were consistent with the amounts of their glycosylated forms (Fig. 1B, lanes 8, 9). Moreover, we detected the sensitivity of these BST-2 variants to HIV-1 Vpu by evaluating their abilities to inhibit wild-type HIV-1. 293T cells were co-transfected with BST-2 variants and pNL4-3, followed by analysis of the cell lysates and released capsid proteins by Western blotting. The results were consistent with Fig. 6A (Fig. 6B).

### Epitope tags beside the N-terminus of CT-truncated BST-2 alter its intracellular trafficking

**Figure 7:**
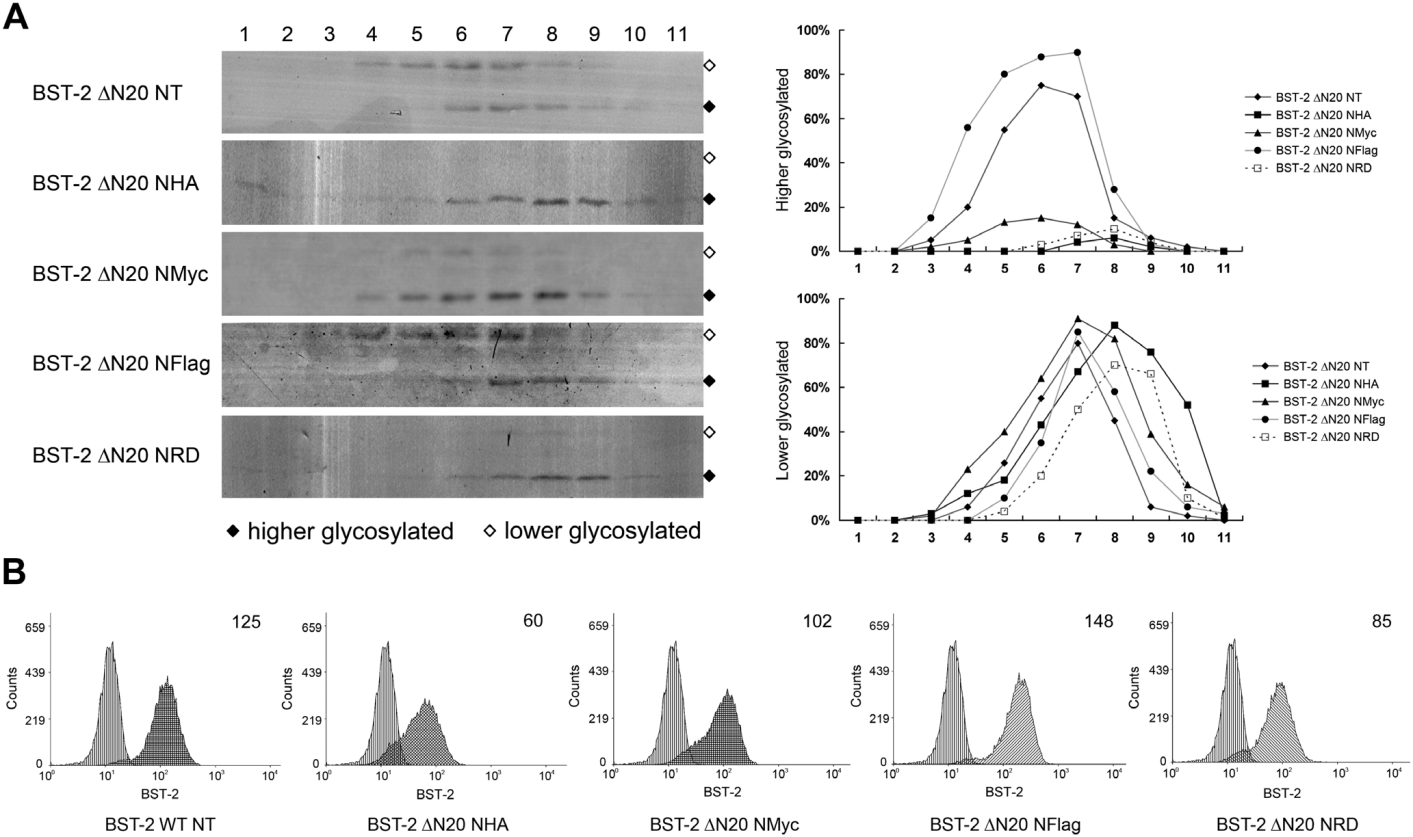
Fusion of different epitope tags to N-terminus of BST-2 ΔN20 results in different effects on intracellular trafficking. (A) 293T cells in 10-cm plates were respectively transfected with BST-2 ΔN20 NT, BST-2 ΔN20 NHA, BST-2 ΔN20 NMyc, BST-2 ΔN20 NFlag, BST-2 ΔN20 NHARD and the cell lysates were analyzed by sucrose gradient ultracentrifugation. Samples were analyzed by Western blotting with an anti-BST-2 pAb. The major bands corresponding to higher and lower glycosylated BST-2 are marked with symbols. The level of glycosylated forms of BST-2 in each sample was respectively quantified and presented in line arts. (B) The cell surface expression of BST-2 ΔN20 NMyc, BST-2 ΔN20 NFlag and BST-2 ΔN20 NHARD were analyzed by the FACS assay as described above in Figure. 5. This experiment was repeated for three times and the most representative data was shown.

To demonstrate if the altered antiviral function related with the abnormal intracellular trafficking, we analyzed the cellular fractionation of BST-2 ΔN20 variants with different tags using sucrose gradient ultracentrifugation. Eleven fractions were collected from the top of the gradient and analyzed by Western blotting. Compared with BST-2 ΔN20 NT, BST-2 ΔN20 NHA and NHARD exhibited as more lower glycosylated forms and mainly existed in fractions with larger density (Fig. 7A). The myc tag also exhibited partial impact. In comparison, the flag tag exhibited no notable impact in this characteristic.

These variants have also been analyzed by FACS assay as described above in Figure. 5 to verify the cell surface expression. The surface level of BST-2 ΔN20 with the NFlag, NMyc and NHARD tags were in that relative order from high to low (Fig. 7B). Notably, NFlag tag merely affects the surface level of BST-2 ΔN20, while NHARD tag impairs the function of BST-2 ΔN20 which performs like NHA tag.

### The hydrophobicity and positive charged residues of the cytosolic region potentially control the intracellular trafficking of BST-2

**Figure 8:**
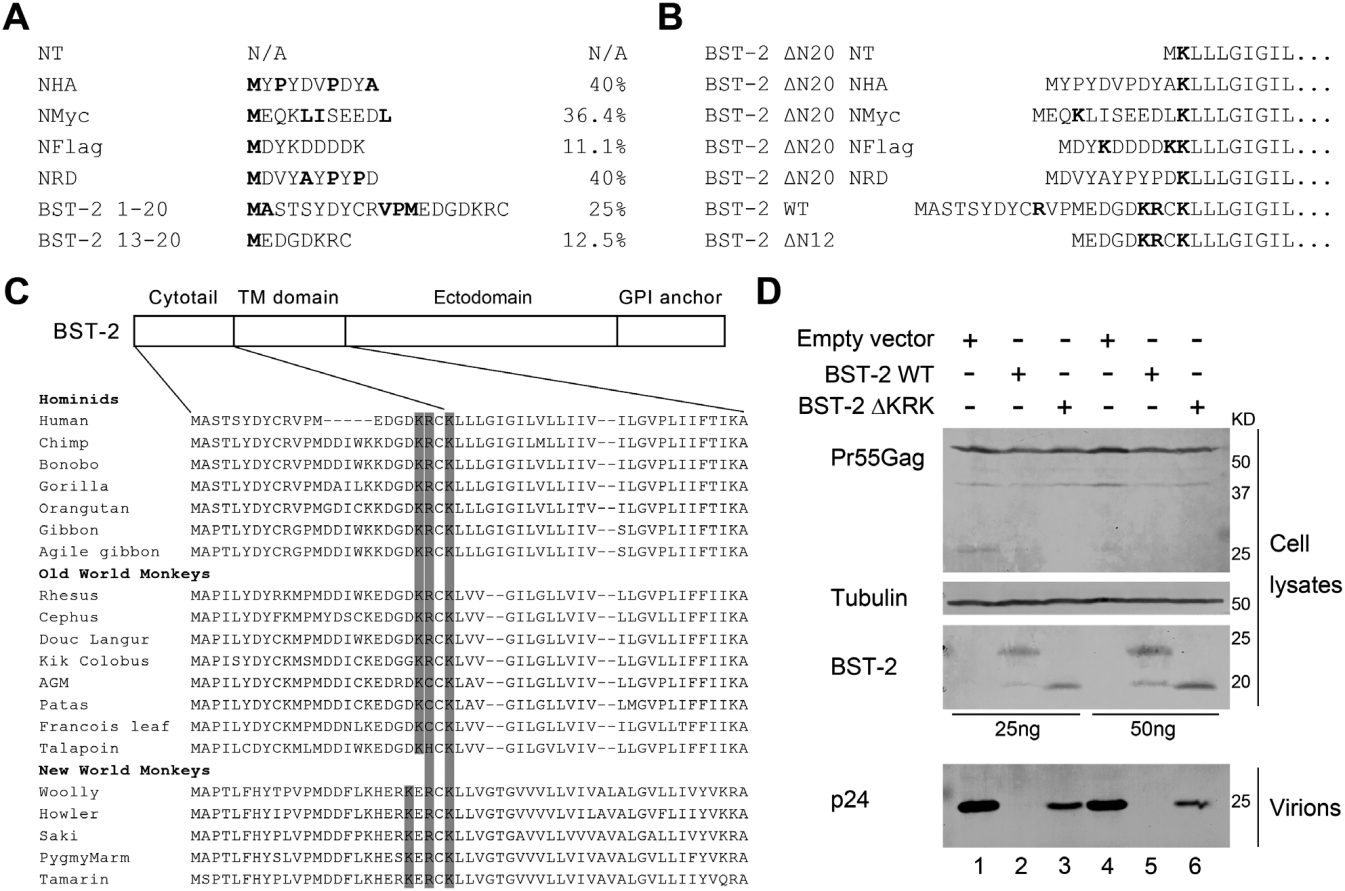
Comparison of epitope tagged N-terminus of BST-2 ΔN20 with its native cytosolic region and orthologues. (A) Analysis of hydrophobicity in N-terminal peptides of BST-2 variants. The hydrophobic residues (M, F, W, V, L, I, P, A) were marked and the percentages were calculated to represent the total hydrophobicity. (B) Analysis of positive charged residues (K, R, H) in N-terminal regions of BST-2 variants. (C) The sequence alignment of BST-2 and its orthologues in the N-terminus. The conserved positive charged residues in the conjunctive region between the cytotail and the transmembrane domain were marked. (D) Indicated amount of BST-2 WT or BST-2 ΔKRK, a mutant with mutated above mentioned positive charged residues was co-transfected with 1 µg of the proviral plasmid pNL4-3 ΔVpu in 293T cells. At 48 h post-transfection, cultured supernatants were ultracentrifuged to concentrate the virus particles. Virions and cell lysates were analyzed by Western blotting.

Next we analyzed basic characteristics of these N-terminal tags and native cytosolic domain of human BST-2, attempting to draw parallels with the virion release restriction. As the cytosolic region usually appears as hydrophilic nature, we wondered if the dysfunction is related to the excessive hydrophobic residue content. The hydrophobic residues (M, F, W, V, L, I, P, A) were marked (Fig. 8A). The percentage of hydrophobic residues in NHA and NHARD is 40%, which is greater than that for NMyc (36.4%) and NFlag (11.1%). While the cytosolic region of BST-2 (BST-2 1–20) and short form BST-2 (BST-2 13–20) is 25% and 12.5%, respectively. Thus, the excessive proportion of hydrophobic residues in the N-terminus may impair the normal trafficking and the antiviral activity of BST-2.

As positive charged residues (K, R, H) play important roles in N-terminal leader peptide which lead the protein trafficking, we further analyzed positive charged residues (K, R, H) in N-terminal regions of BST-2 variants (Fig. 8B). BST-2 ΔN20 NFlag had two Ks in addition to one K of BST-2 ΔN20 NT, while no more such residues existed in NHA and NHARD. In parallel, native BST-2 has three positive charged residues (K18, R19 and K21) in the conjunctive region between the cytotail and the transmembrane domain. Significantly, the sequence alignment of BST-2 and its primate orthologues in the N-terminus revealed that these residues is much conserved in primates (Fig. 8C), suggesting this may be a key feature of BST-2 to control its protein trafficking. To demonstrate that, we mutated these residues of human BST-2, and the mutant BST-2 ΔKRK exhibited impaired function in the glycosylation and antiviral activities (Fig. 8D).

### Alteration of CT or an internal tag in BST-2 affects its ability to activate NF-κB activation

**Figure 9:**
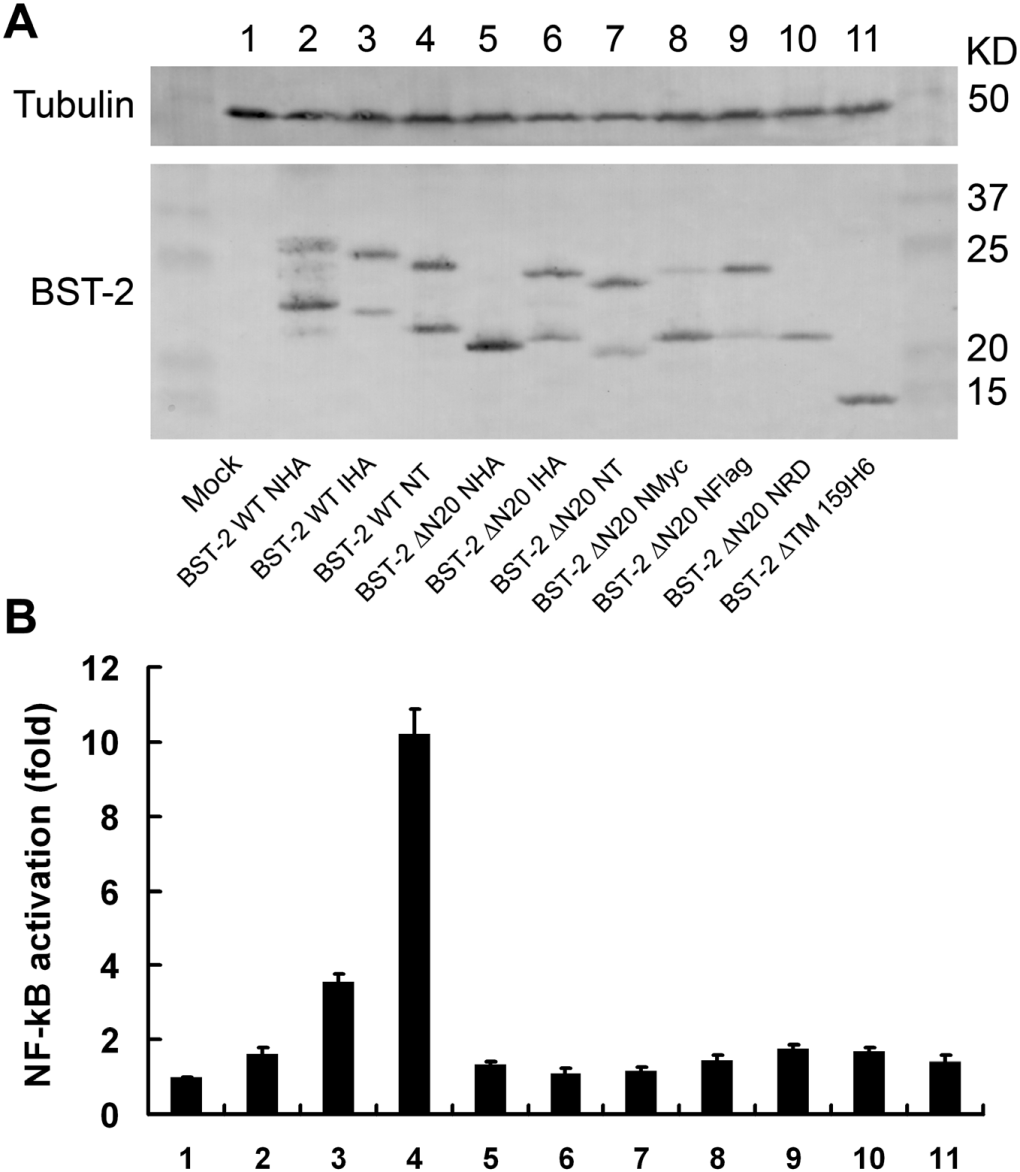
NF-κB activation mediated by BST-2 variants. (A) 293T cells were co-transfected with 25 ng of variant BST-2 expression plasmids, along with 250 ng of pNF-κB-Luc reporter plasmid and 50 ng Renilla luciferase plasmid. After 48 h, cells were lysed and analyzed for luciferase activity. BST-2 expression was analyzed by Western blotting using an anti-BST-2 pAb. (B) The raw luciferase value was normalized to the renilla luciferase value. The data were compared with the mock control and shown as fold of NF-κB activation. The graph was generated from three independent experiments.

Although primarily characterized as a cell intrinsic viral restriction factor, BST-2 has been proposed to have additional activities, including a role in induction of the proinflammatory response regulator NF-κB [25], [26], [27]. To explore if the various mutations in BST-2 affect its ability to activate NF-κB, 293T cells were co-transfected with indicated wild-type or mutated BST-2 expression plasmid and luciferase reporter plasmid pNF-κB-luc along with a Renilla luciferase plasmid as transfection control. The cell lysates were analyzed for BST-2 expression (Fig. 9A) and luciferase activity at 48 h post-transfection. BST-2 WT NT led to NF-κB activation approximately 10-fold over mock control cells transfected with the expression vector alone, while 4-fold activation was led by BST-2 WT IHA. In contrast, the activities of BST-2 WT NHA and all CT-truncated BST-2 were negligible (Fig. 9B). These observations suggest that the CT plays a considerable role in BST-2-induced NF-κB activation and that the N-terminal or internal tag added to BST-2 also affects its function of NF-κB activation.

## Discussion

The predominant mechanism for BST-2-mediated inhibition of viral particle release is the direct tethering of virions to cells. In this process, the TM domain and GPI domain of BST-2 are responsible for binding the cell membrane with the viral membrane [20]. The function of the cytosolic domain has been studied, however, the role of the cytosolic domain in the virion retention is not clear. Based on the current knowledge of feline BST-2, we suspected that the N-terminal CT of human BST-2 would be necessary for its antiviral function. In this study, we constructed a series of wild-type and mutant BST-2 proteins with various internal or N-terminal tags and initially confirmed their expression levels by Western blotting. No notable differential expression was observed among most of BST-2 variants, except that BST-2 N20 NHA expression is relatively stronger than other variants.

In order to understand the contribution of the BST-2 CT to its antiviral activity, we performed virion release assays and observed that the internally HA-tagged and no tag truncated BST-2 proteins showed equivalent levels of activity against Vpu-defective HIV-1 compared with the wild-type BST-2. In addition, BST-2 ΔN20 NHA showed severe impairment of antiviral activity with both Vpu-defective and wild-type HIV-1. This phenomenon demonstrated that the cytoplasmic region of BST-2 contributed quite little to its antiviral function, as even BST-2 protein lacking the entire CT exhibited an enhanced inhibitory effect on wild-type HIV-1 virus, indicating an altered sensitivity to HIV-1 Vpu. These results were partially inconsistent with earlier findings emphasizing the importance of the CT to the antiviral function of BST-2 [4]. Therefore, we further analyzed the mutant used in this former study and found that the HA peptide sequence fused to the N-terminus of the CT-truncated BST-2 ΔN20 eliminated the remaining antiviral function observed with BST-2 ΔN20 NT. These findings suggested that the CT of human BST-2 is functionally unnecessary, at least for its antiviral function. Moreover, the interaction between Vpu and BST-2 may not only depend on their respective TM domains but also require the complete CT of BST-2, which may contain a yet undiscovered region for Vpu binding or for folding of the TM helix. To some extent, our results generally agreed with a former discovery [20], but this current study has indeed provided new detailed information to help further define the structure-function relationship of BST-2.

Based on these observations, the underlying mechanism for the effect of the HA tag on the antiviral function of BST-2 ΔN20 required further investigation. At first, we hypothesized that the N-terminus of this mutant protein was incomplete and was potentially cleaved immediately after translation resulting from the unexpected formation of a leader peptide sequence. However, N-terminal sequencing of the affinity purified protein showed that the HA tag in the N-terminus of this protein was intact, implying that the smaller apparent size of the BST-2 ΔN20 NHA mutant on the SDS-PAGE gel was due to some other reason. The glycosylation analysis indicated that the BST-2 ΔN20 NHA mutant did not undergo N-glycosylation even though it still contained putative N-glycosylation sites. This result implied that the original trafficking pathway of the native protein may undergo unpredictable changes, subsequently leading to the rearrangement of the intracellular distribution and alteration of surface expression.

The immunofluorescence analysis showed that the cellular distribution of BST-2 ΔN20 NHA appeared as dense perinuclear aggregates, which was different from the uniform puncta-like distribution of the other mutants and the wild-type protein. Meanwhile, flow cytometry analysis confirmed that the cell surface level of BST-2 ΔN20 NHA was only a small portion of its total cellular expression. This alteration in cell surface level could most directly explain the dysfunction of the mutant. We speculated that the additional sequence in the N-terminus retained the misfolded protein in the ER primarily and hindered its transit through the secretory pathway. A possible mechanistic explanation is that BST-2 ΔN20 NHA fails to be translocated across the ER membrane. But due to its membrane spanning domain, it retains around the ER membrane in the cytosol as perinuclear clusters. While the control mutant BST-2 ΔTM 159H6 without any spanning domain, totally loses membrane association and becomes diffuse cytosolic. This may be the major difference between BST-2 ΔN20 NHA and ΔTM 159H6. Additionally, we further showed that the expression of BST-2 ΔN20 NHA causes the exaggeration of the ER membranes. This could be induced by the continual aggregation of the newly-synthesized BST-2 proteins at ER membranes, which process may subsequently increase ER capacity and stress. These abnormal processes ultimately caused the impaired trafficking and reduction of cell surface expression of the protein, which was most closely associated with the decline of antiviral activity.

To some extent, the topology of BST-2 ΔN20 NHA is equivalent to the protein with the entire CT domain replaced with an HA tag. Another question that has arisen is whether the lack of membrane binding of this mutant was due to specific residues in the HA sequence. Several BST-2 ΔN20 mutants carrying different commonly used epitope tags were constructed to examine this issue. The virion release assay along with the flow cytometric analysis showed the distinct properties of these mutants. The rearrangement of the HA sequence had little effect on preventing the loss of the normal antiviral function of BST-2. By analyzing the basic characteristics of these peptides, we found that the above phenomenon was potentially related to the ratio of hydrophobic amino acids in the peptide tags. In other words, an increased number of hydrophobic amino acids in the tag was associated with a greater reduction in the protein function. Additionally, as positive charged residues (K, R, H) play important roles in N-terminal leader peptide which lead the protein trafficking, we noticed that the epitope tags which maintain the normal function of BST-2 ΔN20 (Flag and Myc to less extent) posses more of these residues. Futher, we demonstrated that a positive charged motif “KRXK” in the conjunctive region between the cytotail and the transmembrane domain which is conserved in primate BST-2 is important for the protein trafficking.

Interestingly, the C-terminus of the HA tag and the N-terminus of BST-2 ΔN20 form an YXXL sequence, and the YXX*Φ* motif (Y = tyrosine, X = any amino acid, *Φ* = large hydrophobic residue) in the CTs of receptors is known to interact with adaptor complexes [55]. Additionally, HIV-1 Env contains a classical YXX*Φ*clathrin-adaptor protein (AP) complex binding motif [56], which is required for the HIV-2 Env-mediated removal of BST-2 from the cell surface and the enhancement of virion release [38], [57]. These observations suggest that the motif may further enhance the endocytosis of BST-2 and can also be a possible secondary mechanism for the low cell surface level of BST-2 ΔN20 NHA.

The luciferase activity analysis showed that the ability of CT-truncated BST-2 to activate NF-κB was impaired. This loss of function was expected as the determinants of NF-κB activation are currently defined as a tyrosine-based motif (YxY) and an RVP (10–12) motif found within the CT of BST-2. Moreover, BST-2 WT NHA was also deprived of this capacity to activate NF-κB, and that of BST-2 WT IHA was severely weakened. These results implied that the HA tag affects the native conformation of wild-type BST-2, even when it has no influence on the antiviral activity of the protein. These results also confirmed that the BST-2 function of NF-κB activation is distinct from its restriction of virus release. Additionally, these observations highlighted the importance of the choice of tag type and position in functional studies of biological molecules.

In conclusion, our results provided evidence that the host BST-2 protein lacking nearly the entire N-terminal CT still retained antiviral activity against Vpu-defective HIV-1 and could even inhibit wild-type HIV-1 viral particle release. However, the fusion of different peptide labels to the N-terminus of the CT truncated BST-2 protein impaired its antiviral activity to varying degrees. The excessive proportion of hydrophobic amino acids in the N-terminal cytoplasmic peptide may have affected the association of the recombinant BST-2 with lipid membranes such as ER retention. Such impairment in intracellular trafficking influenced the N-glycosylation and reduced localization of the N-terminally tagged truncated BST-2 to the cell surface, eventually weakening its antiviral function. While this manuscript was in preparation, a very recent study in Biology Open by Billcliff and colleagues reported that the cytosolic N-terminus of BST-2 has the potential of a membrane microdomain exclusion motif which mainly regulates protein trafficking [58]. Our observations largely support their study, however, the detailed findings are different. Importantly, our results demonstrated that the CT-truncated BST-2 would be a more powerful antiviral protein than the native factor. Additionally, the alteration of BST-2 CT or an internal tag was found to affect its capability to activate NF-κB. These results provide additional details and implications for the structure-function model of BST-2.

## Supporting Information

Figure S1 **Comparison of BST-2 ΔN12 and ΔN20 variants and the impact of NHA tag.** (A) Indicated amount of BST-2 WT, BST-2 ΔN12 or BST-2 ΔN20 was co-transfected with 1 µg of pNL4-3 ΔVpu in 293T cells. At 48 h post-transfection, cultured supernatants were ultracentrifuged to concentrate the virus particles. Virions and cell lysates were analyzed by Western blotting. (B and C) 50 ng of BST-2 WT NT, BST-2 ΔN12 NT/NHA or BST-2 ΔN20 NT/NHA was co-transfected with 1 µg of pNL4-3 ΔVpu/pNL4-3 WT in 293T cells. Concentrated Virions and cell lysates were analyzed by Western blotting. (D) COS-7 cells transfected with 200 ng of control plasmid VR1012, BST-2 variants expression plasmids were observed by confocal microscopy; blue, cell nucleus; green, BST-2 protein.
(TIF)

Figure S2 **The potential impact of BST-2 ΔN20 NHA to the exaggeration of the ER membranes.** The ER-Tracker staining and Merge images of BST-2 ΔN20 NT and BST-2 ΔN20 NHA. blue, cell nucleus; green, BST-2 protein; red, ER. BST-2 expressing cells were marked with white arrows. Images were taken under a Zeiss LZM710 confocal microscope.
(TIF)
