# Supplementary figures and images for: Epitope Tags beside the N-Terminal Cytoplasmic Tail of Human BST-2 Alter Its Intracellular Trafficking and HIV-1 Restriction
Source: PLoS One. 2014 Oct 27;9(10):e111422. doi: 10.1371/journal.pone.0111422 (PMC4210262; doi:10.1371/journal.pone.0111422)

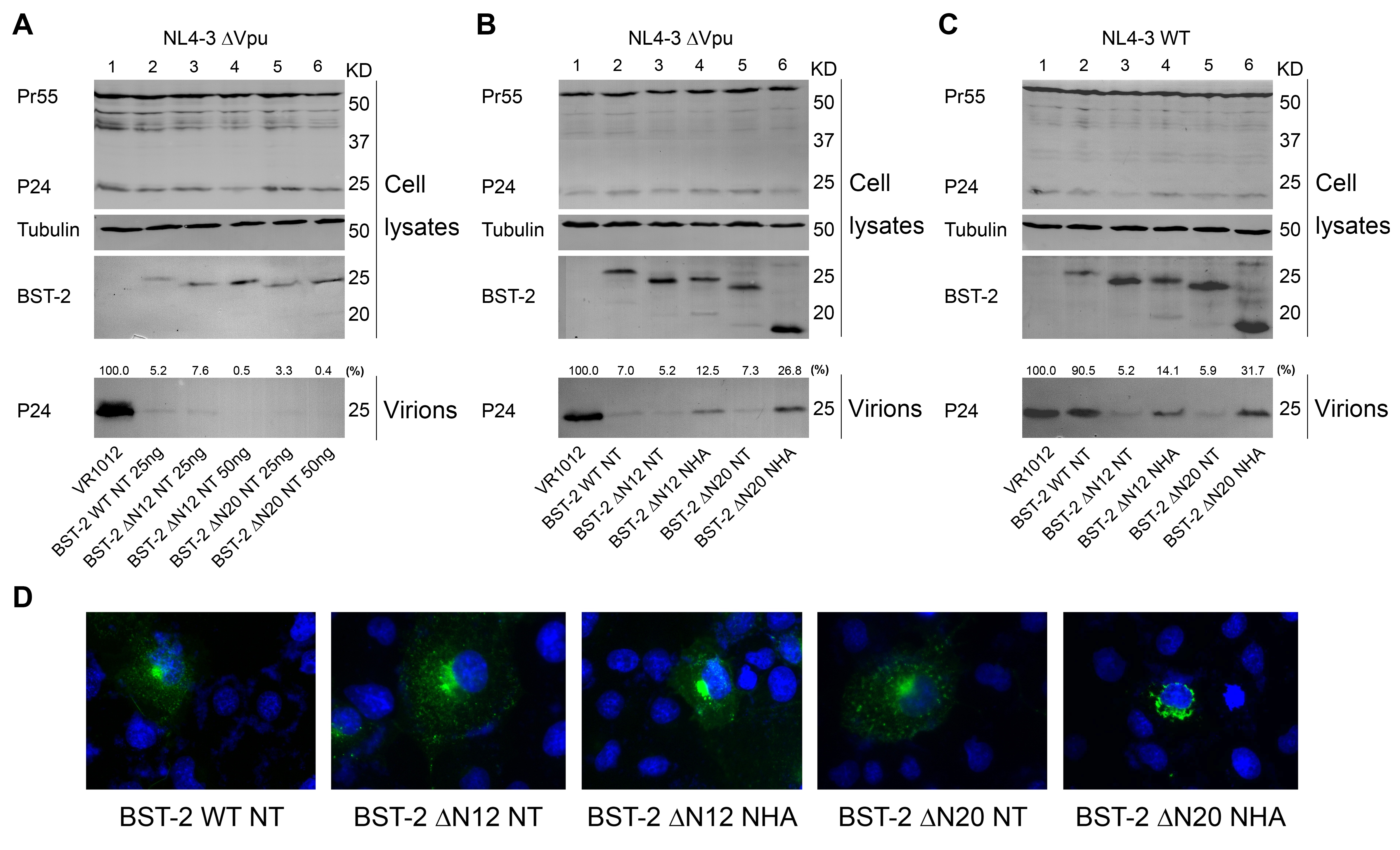

Supplement: Figure S1 — Comparison of BST-2 ΔN12 and ΔN20 variants and the impact of NHA tag. (A) Indicated amount of BST-2 WT, BST-2 ΔN12 or BST-2 ΔN20 was co-transfected with 1 µg of pNL4-3 ΔVpu in 293T cells. At 48 h post-transfection, cultured supernatants were ultracentrifuged to concentrate the virus particles. Virions and cell lysates were analyzed by Western blotting. (B and C) 50 ng of BST-2 WT NT, BST-2 ΔN12 NT/NHA or BST-2 ΔN20 NT/NHA was co-transfected with 1 µg of pNL4-3 ΔVpu/pNL4-3 WT in 293T cells. Concentrated Virions and cell lysates were analyzed by Western blotting. (D) COS-7 cells transfected with 200 ng of control plasmid VR1012, BST-2 variants expression plasmids were observed by confocal microscopy; blue, cell nucleus; green, BST-2 protein. (TIF) [file pone.0111422.s001.tif]

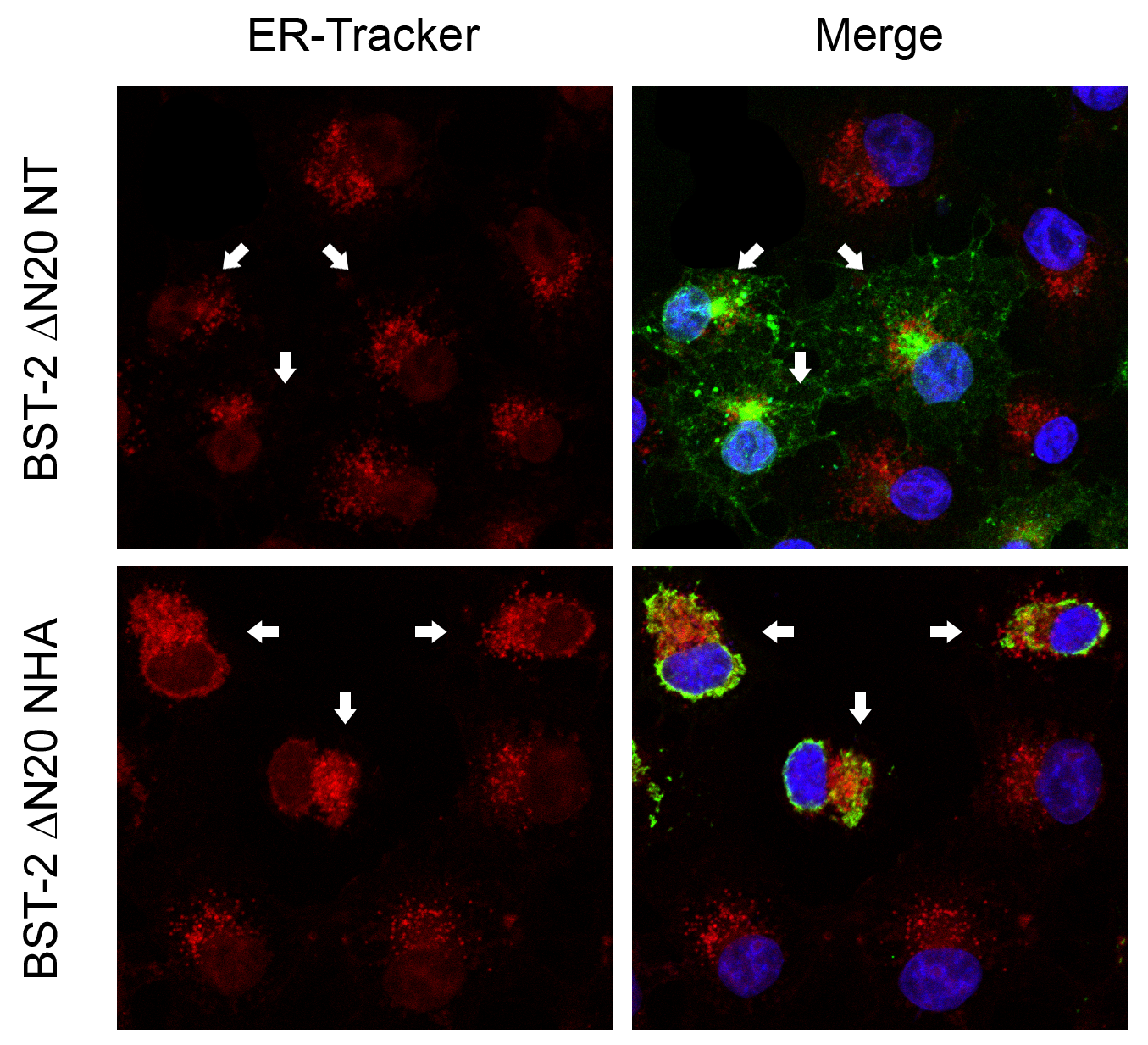

Supplement: Figure S2 — The potential impact of BST-2 ΔN20 NHA to the exaggeration of the ER membranes. The ER-Tracker staining and Merge images of BST-2 ΔN20 NT and BST-2 ΔN20 NHA. blue, cell nucleus; green, BST-2 protein; red, ER. BST-2 expressing cells were marked with white arrows. Images were taken under a Zeiss LZM710 confocal microscope. (TIF) [file pone.0111422.s002.tif]
